# Type 2 Innate Lymphoid Cells Protect against Colorectal Cancer Progression and Predict Improved Patient Survival

**DOI:** 10.3390/cancers13030559

**Published:** 2021-02-01

**Authors:** Qiutong Huang, Nicolas Jacquelot, Adele Preaudet, Soroor Hediyeh-zadeh, Fernando Souza-Fonseca-Guimaraes, Andrew N. J. McKenzie, Philip M. Hansbro, Melissa J. Davis, Lisa A. Mielke, Tracy L. Putoczki, Gabrielle T. Belz

**Affiliations:** 1 Walter and Eliza Hall Institute of Medical Research, Parkville, Melbourne 3052, Australia; huang.q@wehi.edu.au (Q.H.); jacquelot.n@wehi.edu.au (N.J.); preaudet.a@wehi.edu.au (A.P.); hediyehzadeh.s@wehi.edu.au (S.H.-z.); davis.m@wehi.edu.au (M.J.D.); Lisa.Mielke@onjcri.org.au (L.A.M.); putoczki.t@wehi.edu.au (T.L.P.); 2 Department of Medical Biology, University of Melbourne, Parkville, Melbourne 3010, Australia; 3 The University of Queensland Diamantina Institute, 37 Kent Street, Woolloongabba, Brisbane 4102, Australia; f.guimaraes@uq.edu.au; 4 Medical Research Council Laboratory of Molecular Biology, Cambridge CB2 0QH, UK; anm@mrc-lmb.cam.ac.uk; 5 Center for Inflammation, Centenary Institute and the School of Life Sciences, University of Technology Sydney, Sydney 2050, Australia; Philip.Hansbro@uts.edu.au; 6 Department of Clinical Pathology, University of Melbourne, Parkville, Melbourne 3010, Australia; 7 Olivia Newton-John Cancer Research Institute, La Trobe University School of Cancer Medicine, Heidelberg 3084, Australia

**Keywords:** colon cancer, colitis-associated cancer, ILC2, IL-5, IL-13, inflammation

## Abstract

**Simple Summary:**

Colorectal cancer is the second leading cause of cancer-related death worldwide. The immune system plays a key role in controlling tumour onset and development. However, our immune system is complex and includes many different cell types which differently impact colorectal cancer outcomes. In this study, we investigated the function of the specialised type 2 innate lymphoid cells (ILC2) in colorectal cancer development and progression. We found that ILC2 infiltrate colorectal tumours and their presence was associated with reduced tumour burden in mice. In patients, this infiltration correlated with improved overall survival. Collectively, our work reveals that ILC2s are beneficial to colorectal cancer outcomes.

**Abstract:**

Chronic inflammation of the gastrointestinal (GI) tract contributes to colorectal cancer (CRC) progression. While the role of adaptive T cells in CRC is now well established, the role of innate immune cells, specifically innate lymphoid cells (ILCs), is not well understood. To define the role of ILCs in CRC we employed complementary heterotopic and chemically-induced CRC mouse models. We discovered that ILCs were abundant in CRC tumours and contributed to anti-tumour immunity. We focused on ILC2 and showed that ILC2-deficient mice developed a higher tumour burden compared with littermate wild-type controls. We generated an ILC2 gene signature and using machine learning models revealed that CRC patients with a high intratumor ILC2 gene signature had a favourable clinical prognosis. Collectively, our results highlight a critical role for ILC2 in CRC, suggesting a potential new avenue to improve clinical outcomes through ILC2-agonist based therapeutic approaches.

## 1. Introduction

The GI tract is lined with a single layer of epithelial cells that create a physical barrier to assist with the prevention of trillions of microorganisms translocating from the intestinal lumen into the systemic circulation, which could otherwise lead to serious complications, including sepsis. Millions of tissue-resident immune cells patrol the GI tract, making it the largest component of immune surveillance within the body [1]. Consequently, a fine balance between pro- and anti-inflammatory immune responses is required to maintain GI homeostasis [1]. Chronic dysregulation of these immune responses has been shown to contribute to CRC development and progression in mice [2,3], mirroring the observation that inflammatory bowel disease (IBD) patients have an increased risk of CRC development [4].

While the contribution of the adaptive immune response to CRC onset and progression is well described [5,6], much less is known about the role of the innate immune response [7,8]. Innate lymphoid cells (ILCs) comprise a major component of GI inflammation, and have been implicated in murine CRC models [3,9,10] and found within human CRC tumours [11,12]. ILCs are evolutionarily conversed [13], mimicking key functions of adaptive T lymphocytes [14]. They are grouped into five subsets: natural killer (NK) cells, ILC1, ILC2, ILC3 and lymphoid tissue-inducer (LTi) cells based on their developmental program, transcription factors and cytokine expression [15,16].

NK cells and ILC1 express T-bet, produce interferon-(IFN)-γ and granulocyte-macrophage colony-stimulating factor (GM-CSF) [17] and have well-described roles in responses to viral [18] and bacterial infections [19,20]. Similar to the anti-tumourigenic activity described for other tumour types [21,22,23], NK cells can effectively induce cell death in cancer cells, including CRC stem cells and cancer-initiating cells [24]. However, due to the selective pressure imposed by the microenvironment, tumour cells may evade NK cell detection and tumour cell lysis by upregulating MHC-Class I and downregulating NCR ligands to escape NK-cell-mediating killing [24]. An intraepithelial-like ILC1 population has been found to infiltrate human CRC and have the capacity for granzyme and perforin secretion as well as IFN-γ production, making them potential key players in the anti-tumour response [11,12].

ILC3s and LTi cells express the transcription factor RORγt and produce IL-17A and IL-22, cytokines that are essential to the host response to bacterial infections and maintenance of intestinal homeostasis, although their role in wound healing responses can also lead to augmented tumour progression [9,25,26,27,28]. The activity and function of ILC3s are also regulated by IL-23 [29], a cytokine that is produced by activated intestinal dendritic cells and macrophages [30], with IL-23 also shown to promote CRC development [31]. While LTi cells are critical to lymphoid organogenesis, it is not known if they are involved in CRC progression [32].

Our understanding of the role of ILC2s in CRC is limited. ILC2s express RORα and GATA3 and secrete type-2 cytokines [33,34,35], including interleukin-(IL)5, IL-13 and GM-CSF that promote the clearance of helminth and parasitic infections [36,37]. In addition, ILC2s produce amphiregulin, a protein necessary for wound healing and tissue repair [38]. While IL-13 signalling in CRC [39] and other tumour types [40,41] has been associated with a poor prognosis, the role and function of ILC2-derived IL-5 are still poorly understood. IL-5 is critical for the development, recruitment, activation and survival of eosinophils [42,43] which have been associated with anti-tumour responses and favourable CRC outcomes [44,45,46,47]. Thus, ILC2s may convey different prognostic value according to the level of cytokines that they secrete. This complicates the interpretation of the role of ILC2s in CRC.

Here, we utilised complementary CRC murine models to investigate the role of different ILC subsets in CRC progression. We demonstrate that ILC2-deficient mice developed increased tumour burden compared with littermate wild-type animals. Using an ILC2 gene signature, we reveal that patients with an elevated tumour-derived ILC2 gene signature had increased survival, irrespective of patient age, gender, lymph node status or tumour stage. Collectively, our results demonstrate that ILC2s restrain tumour progression and are associated with favourable disease outcomes, highlighting that they are important potential candidates for therapeutic modulation to boost anti-tumour immunity and improve patient survival.

## 2. Results

### 2.1. ILC Are Present in a Murine Model of Colon Cancer and Implicated in Tumour Growth

In order to understand the role of ILCs in CRC, we first established that ILCs contributed to CRC progression. To this end, we generated subcutaneous MC38 murine colorectal carcinoma syngeneic xenografts in wild-type (C57BL/6) mice, T and B cell-deficient *Rag1^−/−^* mice and severely immunocompromised *Rag2^−/−^γc^−/−^* mice, which lack both adaptive and innate lymphoid cells, and compared the tumour growth rates (Figure S1A,B, Table S1). We found that *Rag1^−/−^* and *Rag2^−/−^γc^−/−^* mice had increased tumour growth and size, and reduced survival compared with wild-type controls, in accordance with previous reports [48,49] (Figure S1B, Table S1). However, we also observed that tumours grew even faster in *Rag2^−/−^γc^−/−^* mice compared to *Rag1^−/−^* mice (Figure S1B, Table S1). Thus, while adaptive immune cells exert a strong influence in controlling the growth of MC38 tumour cells, these data suggest a role for the innate compartment of the immune system in the anti-tumour response.

We then determined whether ILCs had the capacity to infiltrate within the MC38 subcutaneous tumour in wild-type (C57BL/6) mice. After 16 days of tumour growth, we found that all ILC subsets, including NK cells, ILC1, ILC2 and ILC3 infiltrate MC38 tumours (Figure S1C) and represented 5–10% of tumour-infiltrating leukocytes (Figure S1C,D). Interestingly, we also observed an increase in the number of all ILC subsets in tumour-draining lymph nodes (dLN) compared with the non-draining lymph nodes (cLN) (Figure S1E–G), suggesting ILC trafficking within the tumour microenvironment. As all ILC subsets had the capacity to infiltrate into the MC38 tumour, we further examined ILCs during the development of colitis-associated cancer (CAC) in the colon microenvironment.

### 2.2. ILCs Are Increased in Inflammation-Associated Colon Cancer

**Figure 1:**
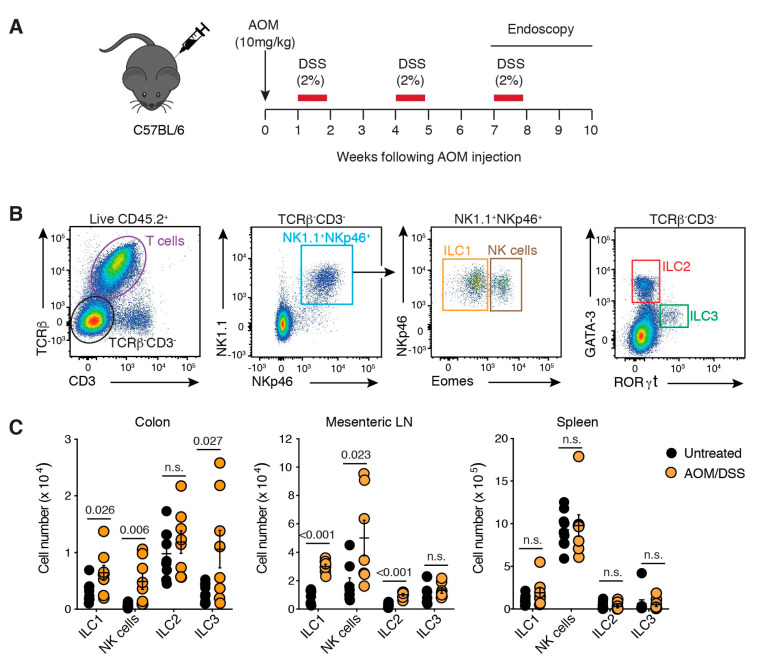
ILCs infiltrate colorectal tumours. (**A**) Schematic illustration of the AOM/DSS treatment protocol. Naïve C57BL/6 mice injected with AOM (10 mg/kg, i.p.) followed by three five-day cycles of 2% (*w/v*) DSS ad libitum in their drinking water separated by two weeks of normal water between each cycle. Tumours developed in the distal colon between 7–10 weeks after the commencement of treatment. (**B**) Flow cytometric analysis of ILCs within lamina propria isolated from the colon. Live CD45.2^+^ lymphocytes were segregated into T cells (TCRβ^+^CD3^+^) and ILCs (TCRβ^−^CD3^−^). ILC subsets were further delimited into NK1.1^+^NKp46^+^ ILC1 (Eomes^−^) and NK cells (Eomes^+^); ILC2s (GATA-3^+^) and ILC3 (RORγt^+^). (**C**) Enumeration of ILC subsets isolated from the colon, mesenteric lymph node (LN) and spleen of untreated and AOM/DSS-treated mice 7–10 weeks after initial treatment. Each dot represents one mouse. Data show mean ± s.e.m of results pooled from four independent experiments (*n* = 2–3 mice/treatment/experiment). Statistics were calculated using an unpaired Student’s *t*-test, *p*-value as indicated. n.s.: not significant.

We next employed a mouse model of CAC to validate our observations in a model more representative of human disease. In the CAC model, animals received the colonic mutagen azoxymethane (AOM), followed by three cycles of the luminal irritant dextran sodium sulphate (DSS) resulting in the formation of tumours in the distal colon (Figure 1A; Figure S2A–C). Tumours in this model are associated with increased inflammatory cell infiltration, which occurs in parallel with the development of regions of adenocarcinoma characterised by disorganised crypt structures (Figure S2D).

We characterised ILC subsets within colons of age- and sex-matched C57BL/6 untreated mice, or mice that developed CAC (Figure 1A,B). Our results demonstrate that although ILC2 numbers were unchanged (Figure 1C), they were the most prevalent ILC subset in the colon at a steady-state (Figure S3, Table S2). In contrast, the number of NK cells, ILC1 and ILC3 increased significantly in tumour-bearing mice (1.9-fold, 3.2-fold and 1.9-fold, respectively; Figure 1C). We also examined the mesenteric lymph node (MLN) and found that ILC1, ILC2 and NK cells accumulated in tumour-bearing mice, while ILC3 numbers did not change, suggesting that ILC3 responses may be localised to the colon (Figure 1C). There were no changes in ILC numbers within the spleen (Figure 1C). These results highlight that ILC responses to CAC are predominantly localised to the GI tract.

### 2.3. The ILC2-Derived Cytokines, IL-5 and IL-13, Are Elevated in CAC

**Figure 2:**
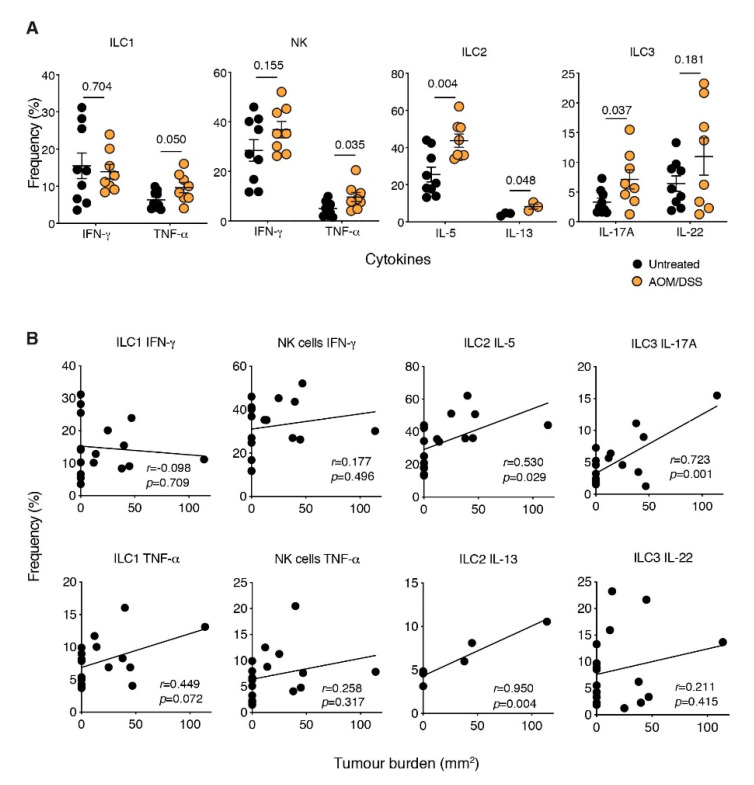
Increased cytokine expression during CAC. (**A**,**B**) Intracellular flow-cytometric analyses of TNF-α, IFN-γ, IL-5, IL-13, IL-17A and IL-22 cytokine production by colonic ILC1s, NK cells, ILC2s and ILC3s after short-term restimulation with PMA, ionomycin in the presence of Golgi Stop and Golgi Plug. Cells were stimulated for 4 h before staining. Frequency and correlation with tumour burden of cytokine-producing ILC isolated from the lamina propria of untreated and AOM DSS-treated mice. Each dot represents one mouse. Data show the mean ± s.e.m. IL-13 data is derived from one experiment and all other data are pooled from four independent experiments (*n* = 2–3 mice/genotype/experiment). Statistical differences were analysed using the Mann–Whitney nonparametric test. The correlation between frequencies and tumour burden was determined using Pearson’s correlation coefficient to calculate *r*. Both the *r* coefficient and *p*-values are indicated.

We next sought to determine if there were changes in the expression level of prototypic ILC cytokines in CAC. Following short-term restimulation of colonic ILC subsets isolated from tumour-bearing CAC mice with phorbol myristate acetate and ionomycin we observed significant increases in TNF-α (ILC1), IL-5 and IL-13 (ILC2) and IL-17 (ILC3) production in tumour-bearing mice (Figure 2A). Among these cytokines, we found that IL-5 expression by colon-derived ILC2s was most highly upregulated in tumour-bearing mice, whereas expression was not altered in the MLN or spleen compared to untreated mice (Figure S4). Interestingly, the frequency of IL-5- and IL-13-expressing ILC2s and IL-17A-producing ILC3s were positively correlated with tumour burden (Figure 2B), suggesting that CAC development may be augmented by the local accumulation of these cytokines in the colon.

### 2.4. Loss of ILC2 Results in Increased Tumour Development in CAC

**Figure 3:**
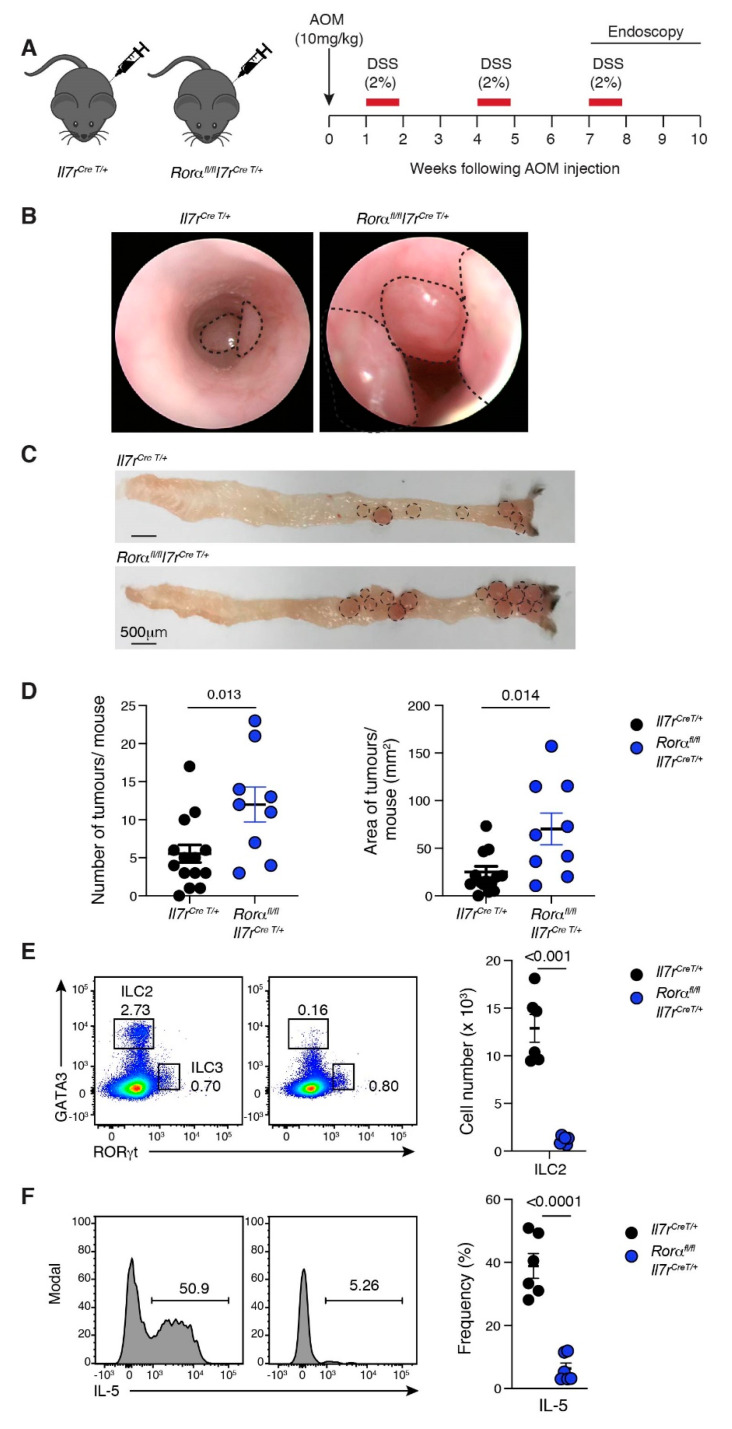
Loss of ILC2 augments CAC. (**A**) Schematic illustration of the AOM/DSS treatment protocol. *Il7r^Cre T/+^* and *Rorα^fl/fl^Il7r^Cre T/+^* mice injected with AOM (10 mg/kg, i.p) followed by three five-day cycles of 2% (*w/v*) DSS ad libitum in their drinking water separated by two weeks of normal water between each cycle. Colonic tumours developed between 7–10 weeks after the commencement of treatment. (**B**) Endoscopic images of AOM/DSS-treated *Il7r^Cre T/+^* and *Rorα^fl/fl^Il7r^Cre T/+^* mice (right panel). Images are representative of two independent experiments (*n* = 9–15 mice/genotype/experiment). Dotted lines indicate colonic tumours. (**C**) Representative whole mount images of colons from *Il7r^Cre T/+^* and *Rorα^fl/fl^Il7r^Cre T/+^* mice treated with AOM+DSS. Images are representative of two to five mice/genotype analysed in two separate experiments. Dotted lines indicate tumour size and location. Scale bar: 500 μm. (**D**) Number (left panel) and area of tumours (mm^2^, right panel) per mouse in *Il7r^CreT/+^* and *Rorα^fl/fl^Il7r^Cre T/+^* mice treated with AOM+DSS. Each dot represents one mouse—closed circle for female. Data show the mean ± s.e.m (*n* = 2–5 mice/genotype/experiment) pooled from two independent experiments. Statistical differences were analysed using the Mann–Whitney nonparametric test. *p*-values are indicated. (**E**) Representative flow cytometric colour plots (left panels) and enumeration (right panels) of ILC2 subsets isolated from the colon of AOM+DSS-treated *Il7r^Cre T/+^* and *Rorα^fl/fl^Il7r^Cre T/+^* mice. Each dot represents one mouse. Data show the mean ± s.e.m. pooled from two independent experiments (*n* = 3 mice/genotype/experiment). Statistical differences were calculated using an unpaired Student’s *t*-test. *p*-values are indicated. (**F**) Representative histograms (left panels) and frequency (right panel) of cytokine-producing ILC2 isolated from the colon of AOM+DSS-treated *Il7r^Cre T/+^* and *Rorα^fl/fl^Il7r^Cre T/+^* mice. Each dot represents one mouse. Data show the mean ± s.e.m. pooled from two independent experiments (*n* = 3 mice/genotype/experiment). Statistical differences were calculated using an unpaired Students *t*-test, *p*-values are indicated.

To determine whether ILC2s have a role in CAC, we crossed the *Rorα^fl/fl^* mouse strain with the *IL-7R^Cre/+^* strain to inactivate the *Rorα* gene by Cre recombinase in IL-7R expressing cells [35] (Figure 3A). *Rorα* is necessary for ILC2s development [35] and is highly expressed in mature ILC2s across all organs, including GI ILC2s [50]. Age- and gender-matched *Rorα^fl/fl^Il7r^CreT/+^* and littermate control *Rorα^+/+^Il7r^CreT/+^* mice underwent the CAC protocol, and tumour development was monitored by endoscopy (Figure 3A,B). While both genotypes developed macroscopic tumours at the mid and distal regions of the colon (Figure 3C), the *Rorα^fl/fl^Il7r^CreT/+^* mice had more and significantly larger tumours (Figure 3B–D). Collectively, these results indicate that the *Rorα* expressing IL-7R-positive immune cells limit CRC progression, as their loss augments tumour growth.

In order to confirm that ROR*α* deletion results in ablation of ILC2s, we analysed the number and function of T cells and ILCs within the colons of untreated (Figure S5) *Rorα^fl/fl^Il7r^CreT/+^* and *Rorα^+/+^Il7r^CreT/+^* mice, compared to tumour-bearing mice (Figure S6 and Figure 3E,F). We observed no changes in the cell numbers of NK cells, ILC3, CD4^+^ and CD8^+^ T cells or their capacity to produce subset-specific cytokines at either a steady-state or during CAC development (Figures S5 and S6). We did, however, find a significant loss of ILC2s, consistent with earlier reports [36,51] (Figure 3E). ROR*α*-deficient mice also showed a reduction in ILC1 numbers within the colon. However, ILC1-deficient *Mcl1^fl/fl^NCR^creT/+^* mice treated with AOM+DSS developed a comparable tumour burden to C57BL6 mice (Figure S7), indicating no protective role in CAC. Collectively, our data demonstrate that ILC2 play a crucial role in preventing CAC tumour progression. Of the few remaining ILC2s in ROR*α*-deficient mice, there was also a reduction in IL-5-producing cells within the colon (Figure 3E,F and Figure S5), suggesting that ILC2-derived cytokines may suppress CRC progression.

### 2.5. An ILC2 Gene Signature Is Associated with Increased Overall Survival in CRC

**Figure 4:**
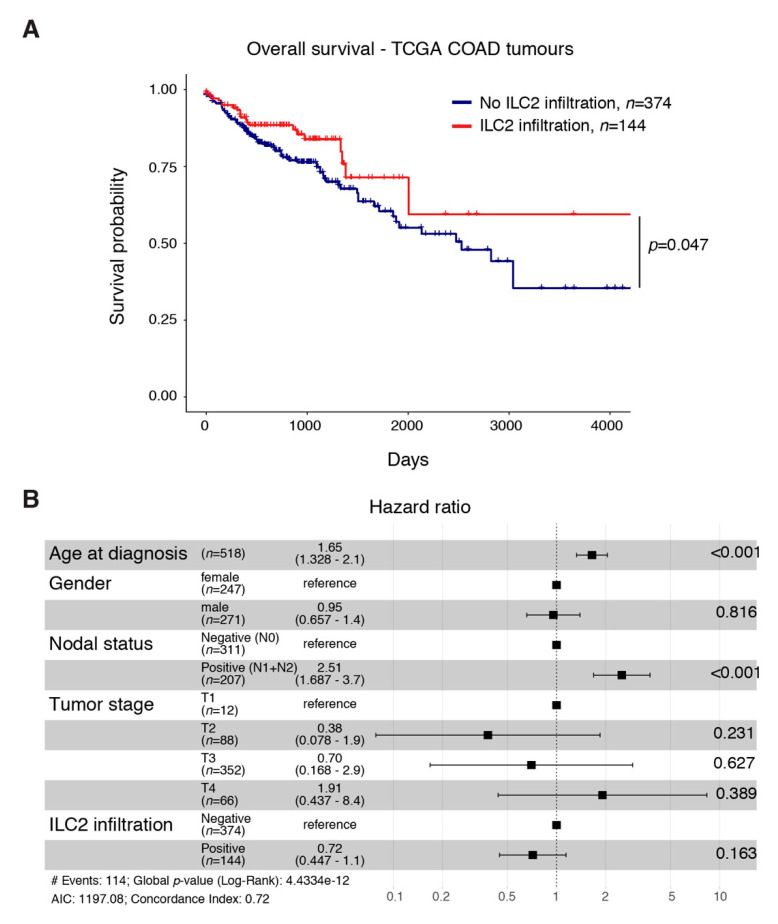
An ILC2 gene signature identifier is associated with improved overall survival for CRC patients. (**A**) Analysis of the impact of tumour ILC2 infiltration on colorectal cancer patient survival using the publicly available TCGA database. (**B**) Multivariate analyses showing hazard ratio of the impact of the age at diagnosis, gender, the presence of metastatic lymph nodes, tumour stage and ILC2 infiltration probability on CRC patient survival.

In order to ascertain the potential relevance of ILC2 and their associated cytokines for the clinic, we generated an ILC2 gene signature using machine learning (Table S3). This gene signature notably includes GATA3 and KLRG1, two genes highly expressed by ILC2 (Table S3). We applied this signature to human colorectal adenocarcinoma RNAseq datasets available within the TCGA database [52] and found that patients with an elevated ILC2 gene signature, indicative of high ILC2 infiltration, had increased overall survival compared with patients with low ILC2 infiltration (Figure 4A). Using multivariate analyses, we found that the favourable prognosis conferred by a high tumour ILC2 gene signature was independent of other covariates including age at diagnosis, sex, the presence of metastatic lymph nodes or the stage of the disease already shown to influence CRC prognosis [6,53,54]. While the age of CRC patients (hazard ratio: 1.65, range 1.328–2.1) and the presence of tumour-invaded lymph nodes (hazard ratio: 2.51, range 1.687–3.7) both significantly negatively impacted cancer prognosis, the presence of ILC2 in tumours (hazard ratio: 0.72, range 0.447–1.1) was independently associated with a better outcome (Figure 4B). Our results suggest that a high ILC2 gene signature may be a useful clinical tool to inform patient treatment strategies in the future.

## 3. Discussion

ILCs were originally discovered over 40 years ago, however, the diversity of the ILC family members has only recently been recognised [15], and the unique features they contribute to the immune response and disease outcomes are only beginning to be unravelled. ILCs respond rapidly during the initial GI immune response to a breach in epithelial barrier integrity and communicate to the adaptive arm of the immune response [55]. However, the role of ILC family members in CRC appears to be divergent, with NK cells required for anti-tumour immunity [12,24], while ILC1 and ILC3 are commonly linked to the promotion of CRC [9,21,29]. Here we show that ILC2s are the predominant ILC member in the inflamed colon of tumour-bearing mice and demonstrate that ILC2s have a role in the restraint of tumour progression.

IL-33 and IL-25 are two well-known ILC2 stimulatory cytokines that are released by damaged epithelial cells. These cytokines induce tissue repair, in part, by activating ILC2s, which have been reported to be important to maintain intestinal tissue integrity [56]. In our study, we found that ILC2s infiltrate CRC tumours and produced higher levels of cytokines than was found in ILC2 from naïve mice and this potentially drove anti-tumour responses. These observations suggest that these tumour-infiltrating ILC2s are likely activated by locally produced ligands such as IL-33 and IL-25 and play a protective role in CRC progression. However, these ligands also have the capacity to promote epithelial cell growth and regulatory T cell activation that can lead to increased tumour development and progression [57,58]. Thus, during the development of tumour cells in the intestinal mucosa, competition for cytokine availability between different cell types can occur and may drive divergent CRC outcomes. This may explain some of the conflicting results previously described [57,59].

The downstream mechanisms of ILC2-mediated protection during CRC are not yet known. Single-cell RNAseq of ILCs during the early and late stages of CRC development in mice identified changes in ILC phenotype and function as tumours developed [3]. In particular, the PD-1^−^ ILC2 subset present during the early stages of CRC was replaced by a predominantly PD-1^+^ ILC2 subset that was capable of promoting tumour development [3]. These observations suggest that the role of ILC2s may change as tumours progress. Recently, it has been shown that the ILC2-derived cytokines, IL-5 and GM-CSF, both have the capacity to control tumour development as the genetic ablation of either IL-5 or GM-CSF increased the tumour burden in the MC38 murine CRC model, while overproduction of IL-5 facilitated protection against tumour development [45]. The protection against the development of CRC was thought to be mediated by eosinophils that depend on IL-5 and GM-CSF to drive migration to the tumour microenvironment, which in turn, promotes Th1 cell responses against tumours [45]. Our findings identify ILC2s as the main IL-5 producer in the colon tumour microenvironment, suggesting that ILC2s may be a key player in mediating tumour protection through the activation of eosinophils, although this remains to be established. We also found increased IL-13 expression by colonic ILC2s isolated from CRC-bearing mice. IL-13-expressing ILC2s are often associated with increased MDSC differentiation and pro-tumorigenic functions in bladder cancer [41], prostate cancer and acute promyelocytic leukemia [40]. RORα-deficient mice would also have reduced IL-13 production due to the absence of ILC2s but it is not known whether this has also contributed to the increase in tumour burden.

Catalogues of the immune cell infiltrate in different genetic and molecular subtypes of CRC are emerging. This information will contribute to the evolution of immunotherapy to treat CRC, beyond the current opportunity for microsatellite instability high/deficient mismatch repair (dMMR-MSI-h) patients [60] and may alter the approach to standard chemotherapy for other patient sub-populations. There is the potential to identify patients who will benefit from targeted boosting of certain immune populations. Thus, an increased definition of the tumour immune infiltrate and an understanding of its prognostic value is urgently needed. As a diagnostic criterion, the tumour immune response and its composition is still largely disregarded in CRC. This is in sharp contrast to the recent findings reported by the Immunoscore^®^ team whose findings strongly advocate for the quantification of CD3^+^ and CD8^+^ T cell infiltration in colorectal cancer [5,6] as prognostic markers to better define the risk of recurrence and survival, and to better guide clinicians in decisions surrounding patient treatment [61,62]. This worldwide taskforce demonstrated that the quality of the immune response in CRC clearly matters and could serve as a strong prognostic indicator for stratifying patients. These analyses pave the way for understanding the contribution of different immune cell subsets such as ILC2s in CRC prognosis and treatment. In the interim, gene signatures that can predict patient outcome are becoming increasingly important in clinical decision making. Since the frequency of ILCs within the tumour microenvironment is low compared with adaptive immune cells, we generated an ILC2 expression signature, which revealed that ILC2s confer a positive clinical outcome, which aligned with our findings in vivo using murine models. Thus, well-modelled machine learning approaches offer resolution beyond the frequency of cell type infiltration to uncover genetic networks and cell types that may have applications in identifying novel links between the infiltration of rare immune cells and patient prognosis.

Collectively, our study has revealed ILC2s as an immune cell subset, associated with favourable CRC outcomes. Further investigation will be important to uncover the key stimuli together with the cellular and molecular mechanisms that drive ILC2 anti-tumour function in CRC. Further studies are now warranted to confirm these findings aiming at determining new ILC2-related targets that might be used in combination with current therapeutic regimens.

## 4. Materials and Methods

### 4.1. Mice

*Rag1^−/−^* and *Rag2^−/−^γc^−/−^* [63], *Rorα^fl/fl^* [35] and *Il7r^Cre^* [64] mice were maintained on a C57BL/6 (Ly5.2) background (originally derived from the Jackson Laboratory) and have been described previously. *Rorα^fl/fl^* mice were crossed to the *Il7r^Cre^* strain to generate the *Rorα^fl/fl^Il7r^Cre^* line that lacks Rorα expression in all lymphocytes [36]. C57BL/6 mice were bred and maintained in specific pathogen free conditions at the Walter and Eliza Hall Institute of Medical Research. Both females and males were used at 6–12 weeks old. Animals were used according to the guidelines of the Australian code for the care and use of animals of the National Health and Medical Research Council of Australia. Experimental procedures were approved by the Animal Ethics Committee of the Walter and Eliza Hall Institute of Medical Research (AEC 2017.037 and 2018.008).

### 4.2. Cell Culture and Xenograft Establishment

The MC38 colorectal cancer cell line was maintained at 37 °C, 5% CO_2_ in complete RPMI-1640 media containing 10% heat-inactivated foetal calf serum (FCS), 1 mM L-Glutamine, 50 mM β-mercaptoethanol, 100 U/mL penicillin, and 100 mg/mL streptomycin. Cells routinely tested negative for *Mycoplasma*. The right flank of mice was shaved and 5 × 10^5^ MC38 cells resuspended in sterile PBS was injected subcutaneously. Tumour size was measured routinely using callipers every two to three days until ethical endpoint. Tumour area was calculated as the length x width of the tumour.

### 4.3. Isolation of Lymphoid Cells

Intestinal tissues: Single-cell suspensions of lymphocytes were isolated from the stomach, small intestine, caecum and colon following incubation for 40 min at 37 °C in Ca^2+^- and Mg^2+^-free Hanks media containing 2% FCS and 5 mM EDTA with gentle shaking to remove intestinal epithelial cells [27,65]. The supernatant was discarded and tissues were then incubated with gentle shaking in 1 mg/mL (*w/v*) collagenase type IV (Worthington, Lakewood, NJ, USA), 200 μg/mL DNase I (Roche, Basel, Switzerland) and 4 U/mL Dispase (Sigma, Darmstadt, Germany) in RPMI-1640 + 2% (*v/v*) heat-inactivated foetal calf serum (FCS) for 45 min at 37 °C. Preparations were filtered and mononuclear cells were isolated by centrifugation on a 40–80% Percoll gradient. Lymphocytes were recovered from the interface and washed twice.

Spleen and lymph nodes: Single-cell suspensions were generated from lymph nodes and spleen by gently dissociating tissues using 70 μm filters. Red blood cells were lysed from the spleen using Red Cell Removal Buffer and washed with PBS prior to antibody staining.

Tumours: Tumours were weighed, minced and incubated with gentle shaking in 1 mg/mL (*w/v*) collagenase type IV (Worthington, Lakewood, NJ, USA) and 0.05 mg/mL DNase I (Roche, Basel, Switzerland) in RPMI-1640 for 45 min at 37 °C. Preparations were filtered and washed in PBS before resuspension in FACS Buffer for flow-cytometry staining.

### 4.4. Flow Cytometry

Single-cell suspensions were stained with the following antibodies: TCRβ (H57-597), NKp46 (29A1.4), NK1.1 (PK136) and fixable viability dye from eBioscience (San Diego, CA, USA); and CD4 (GK1.5), CD8 (53-6.7), CD19 (ID3), CD3ε (145-2C11), CD45 (30-F11), CD45.2 (104), CD90.2 (30-H12) and CD49a (Ha31/8) from BD Biosciences (Franklin Lakes, NJ, USA). Fixation and intracellular staining were performed using the Transcription Factor Staining Buffer Set (eBioscience) and antibodies against GATA-3 (TWAJ, eBioscience), RORγt (Q31-378, BD Biosciences) and EOMES (Dan11mag, eBioscience). Cytokine expression was determined by restimulation of cells isolated by digestion from the colon tissue in the presence of 100 ng/mL phorbol-12-myristate-13-acetate (PMA), 100 ng/mL ionomycin and 10μg/mL GolgiPlug™ and GolgiStop™ (BD Biosciences) in complete RPMI-1640 media (containing 10% heat-inactivated FCS, 1 mM L-glutamine, 100 U/mL penicillin, 100 μg/mL streptomycin and 50 µM β-mercaptoethanol) for 4 h. Cells were then fixed and stained for intracellular cytokines IFN-γ (XMG1.2, BD Pharmigen), TNF-α (Mab11, BD Pharmigen), IL-5 (TRFK5, eBioscience), IL-13 (eBio13A, eBioscience), IL-17A (TC11-18H10.1, BioLegend) and IL-22 (IL22JOP, eBioscience). Cells were analysed using a Fortessa X20 (BD Biosciences) and FlowJo software (Ashland, OR, USA) was used for the analysis.

### 4.5. Colitis-Associated Colon Cancer

Azoxymethane (AOM) was injected intraperitoneally (10 mg/km, Sigma, Darmstadt, Germany) and one week later dextran sodium sulphate (DSS) (molecular mass 36,000–50,000 Da; MP Biomedicals, Santa Ana, CA, USA) was added into drinking water 2% (*w/v*) *ad libitum* for five days followed by two weeks of regular water. This was followed by two additional rounds of treatment with DSS, for a total of three cycles. Tumours at the distal colon were monitored via endomicroscopy using established methods [66] after the second and third round of DSS treatment. Tumour burden, indicated by the total number of tumours and the total area of tumours, was determined on autopsy.

### 4.6. Histology

Tissues were fixed in 10% neutral buffered formalin and processed for wax embedding and routine Hematoxylin and Eosin (H&E) staining. Slides of tissues were scanned in an Aperio and analysed with the ImageScope v11.2.0.780 software (Leica Microsystems Pty Ltd., Wetzlar, Germany)

### 4.7. Training an Intestinal ILC2 Classifier

A classifier for gut ILC2 phenotype was developed using RNAseq data from wild-type mouse ILCs extracted from small intestine lamina propria [67], available on Gene Expression Omnibus (GEO) through accession number GSE85154. Raw counts were downloaded from the corresponding GSE accession, logCPM transformed and TMM normalised [68] using edgeR [69]. A set of 241 genes were selected from a panel of 700 immune genes overlapping the top 1000 highly variable genes in single-cell transcriptome profiles of ILCs extracted from tonsils (GSE70580) [70]. An extreme gradient boosting classifier [71] was trained to 70% of the data using linear boosting (booster = “gblinear”) with a binary logistic loss function (objective = “binary:logistic”), three rounds of iteration (nrounds = 3) and a moderate L1 penalty of 0.05 (alpha = 0.05). The classifier was tested on the remaining 30% of the dataset.

### 4.8. Quantification of Infiltrating ILC2 in the TCGA COAD Cohort Using the Intestinal ILC2 Classifier

Raw RNA sequencing data for TCGA COAD tumours and clinical annotation were downloaded using TCGAbiolinks Bioconductor package [72]. RNAseq counts were logCPM transformed and TMM normalised as described earlier. The classifier was applied to logCPM TCGA COAD data (*n* = 521) to compute a confidence score for the presence of infiltrating ILC2 for each patient. Since the test data, the TCGA COAD cohort, is inherently different and more heterogeneous than the training data, the confidence scores are likely to be over-estimated. We, therefore, computed the probability that model predictions can be wrong by measuring similarities between TCGA RNAseq profiles and class centroids in the training data. For each sample in TCGA, we computed the Euclidean distance between the gene expression profile of the sample and class centroids in the training data, based on the 241 genes included in the model. We then computed the similarity of patient profiles to the training data by applying a radial basis function (RBF) kernel to Euclidean distances. We considered any sample in TCGA with prediction confidence greater than 20% and more than 90% similarity to the gene expression distribution in the training data as confident ILC2 infiltrating samples, resulting in 144 identifications (i.e., 144 patients with gut ILC2 infiltration). A Kaplan–Meier plot and survival analysis were done using survival [73] and survminer [74] R packages based on 518 TCGA COAD patient samples with valid survival data.

### 4.9. Statistical Analysis

All statistical analyses were performed with GraphPad Prism software (Version 7.0 or 8.0, GraphPad Software, San Diego, CA, USA). An unpaired two-tailed Student’s *t*-test was used for pairwise comparisons. The Mann–Whitney test was used to determine the statistical significance of tumour number and burden between either the AOM+DSS treated and untreated group or between mice with different genotypes. Data are shown as the mean ± standard error of the mean (s.e.m). The correlation between cell counts and tumour burden was determined using Pearson’s correlation coefficient to calculate *r*. Tumour growth experiments were analysed with TumGrowth software (https://kroemerlab.shinyapps.io/TumGrowth/) [75] with default settings at the exception of the original tumour measurements that were log-transformed before linear mixed-effect modelling. Cross-sectional analyses were analysed using ANOVA and Holms adjusted *p*-values are indicated. Cox proportional hazards modelling with Holms adjustments were applied when assessing the impact of the genotype on mouse survival. *p*-values ≤ 0.05 were considered statistically significant. All experiments were performed at least two times with similar results obtained for each time.

## 5. Conclusions

Colorectal cancer is associated with an increase in cytokine-producing ILC2s in the colon and these cells are protective against the development of colorectal cancer. Furthermore, an ILC2 gene signature in patient samples is associated with increased survival. This is a novel finding, highlighting the protective role of ILC2s in CRC and is a promising new diagnostic tool to help identify patients with a higher probability of survival.

## Data Availability

Data is contained within the article or supplementary material and may be accessible upon reasonable request to G.T.B.

## Supplementary Materials

The following are available online at https://www.mdpi.com/2072-6694/13/3/559/s1, Figure S1: Enumeration of ILC subsets in MC38 tumour-bearing mice, Figure S2: Incidence of CRC tumours in AOM DSS-treated mice, Figure S3: ILC composition within the intestinal tract, mesenteric lymph nodes and spleen of untreated C57BL/6 mice, Figure S4: The ILC cytokine production in spleen and mesenteric lymph nodes is not influenced during CAC progression, Figure S5: Deletion of RORα results in loss of ILC1 and ILC2 but not T cells or other ILC subsets at steady-state, Figure S6: Deletion of RORα results in loss of ILC1 but not cytokine production or other lymphoid subsets during CAC, Figure S7: Loss of ILC1 and NK cells does not impact CAC development, Table S1: Longitudinal, cross-sectional and survival analyses from MC38 tumour-bearing C67BL6, Rag1^−/−^ and Rag2^−/−^γc^−/−^ for TumGrowth analysis, Table S2: ANOVA analysis of ILC subsets in gastrointestinal organs at steady-state, Table S3: ILC2 gene signature.
