# Supplementary material for: Type 2 Innate Lymphoid Cells Protect against Colorectal Cancer Progression and Predict Improved Patient Survival
Source: Cancers (Basel). 2021 Feb 1;13(3):559. doi: 10.3390/cancers13030559 (PMC7867134; doi:10.3390/cancers13030559)
Supplement: Supplementary file 1 [file cancers-13-00559-s001.pdf]

cancers

MDPI

Type 2 Innate Lymphoid Cells Protect against Colorectal Cancer Progression and Predict Improved Patient Survival

Qiutong Huang, Nicolas Jacquelot, Adele Preaudet, Soroor Hediyeh-zadeh, Fernando Souza-Fonseca-Guimaraes, Andrew N.J. McKenzie, Philip M. Hansbro, Melissa J. Davis, Lisa A. Mielke, Tracy L. Putoczki and Gabrielle T. Belz

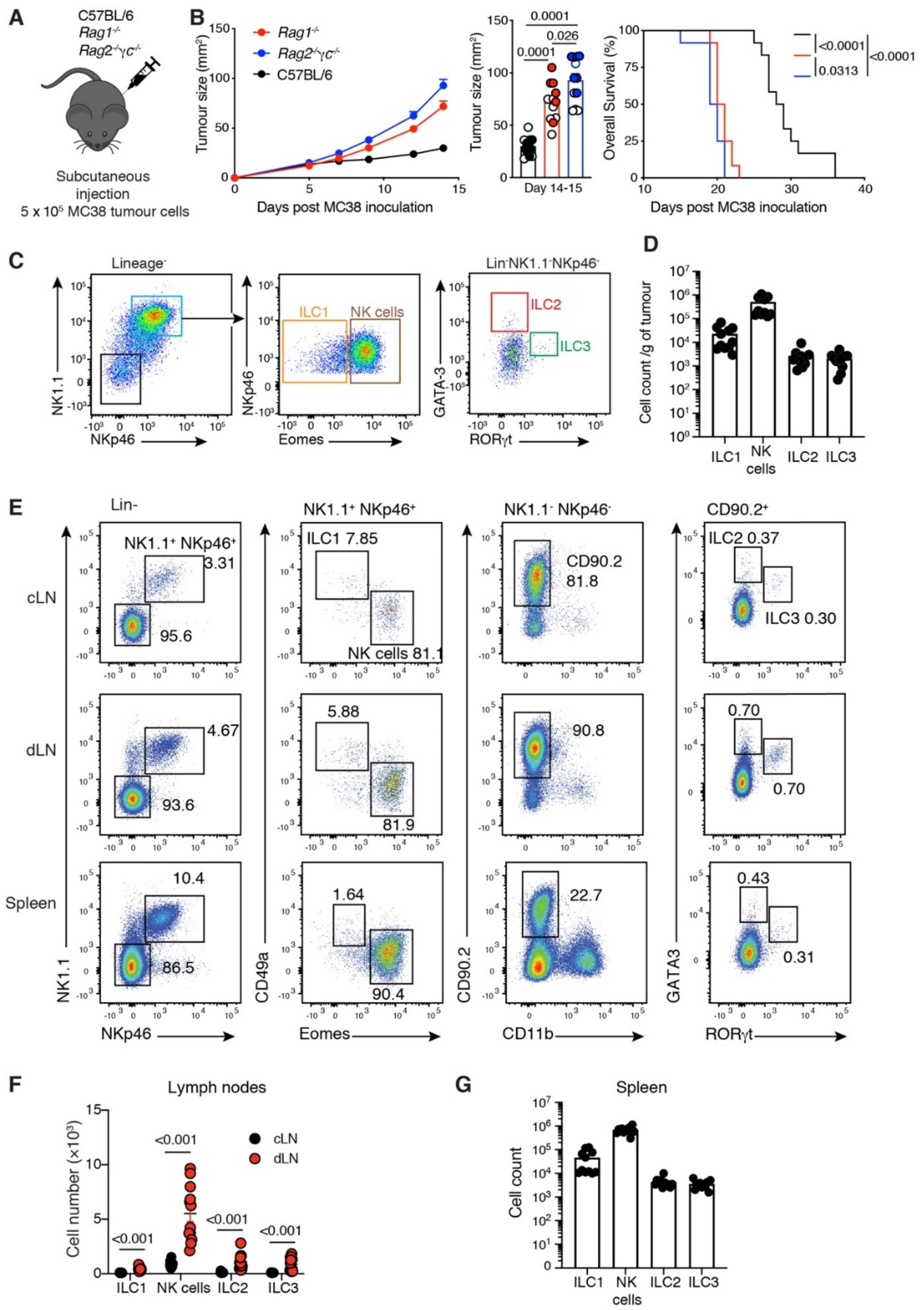

Figure S1. Enumeration of ILC subsets in MC38 tumour-bearing mice. A. Experimental approach to induction of tumours in immunocompetent C57BL/6, *Rag1*$^{-/-}$ (deficient in adaptive immune cells) and *Rag2*$^{-/-}\gamma c^{-/-}$ (deficient in both innate and adaptive immune cells). **B**. C57BL/6, *Rag1*$^{-/-}$ and *Rag2*$^{-/-}\gamma c^{-/-}$ mice were inoculated subcutaneously with MC38 tumour cells. Tumour growth curves over time (left panel), tumour size at day 14-15 post tumour cell inoculation (middle panel) and survival (right panel) are shown. Each dot represents one mouse, closed circle for females, open circles for males. Data show mean ± s.e.m and are pooled from 2 independent experiments with 6 mice/group/experiment (C57BL/6, *n*=12 mice; *Rag1*$^{-/-}$, *n*=12 mice; *Rag2*$^{-/-}\gamma c^{-/-}$, *n*=12 mice). Analyses were performed using the TumGrowth software. **C-G**. Flow cytometric analyses of tumour-infiltrating ILCs. **C**. Representative gating strategies are shown for identification of NK cells, ILC1, ILC2 and ILC3. **D**. Enumeration of tumour-infiltrating ILC isolated at day 16 post MC38 cell inoculation of tumour-bearing C57BL/6 mice. Each dot represents one mouse, closed circle for females. Data show mean ± s.e.m and are pooled from 2 independent experiments with 6 mice/group/experiment. Live NK cells were identified as follow: $CD45^+CD3^-TCR\beta^-CD19^-$ $NK1.1^+NKp46^+Eomes^+$; ILC1 as $NK1.1^+NKp46^+Eomes^-$; ILC2 as $NK1.1^-NKp46^-CD90.2^+CD49a^-CD11b^-GATA3^+$; and ILC3 as $NK1.1^-NKp46^-CD90.2^+CD49a^-CD11b^-ROR\gamma t^+$. **E**. Representative gating strategy to identify ILCs in contralateral lymph nodes (cLN), tumour-draining lymph nodes (dLN), and spleen. Live NK cells were identified as follow: $CD45^+CD3^-$ $TCR\beta^-CD19^-$ $NK1.1^+NKp46^+Eomes^+$; ILC1 as $NK1.1^+NKp46^+Eomes^-$; ILC2 as $NK1.1^-NKp46^-CD90.2^+CD49a^-CD11b^-GATA3^+$; and ILC3 as $NK1.1^-NKp46^-CD90.2^+CD49a^-CD11b^-ROR\gamma t^+$. **F**-**G**. Enumeration of ILC1, NK cells, ILC2 and ILC3 isolated from (cLN), (dLN) (**F**) and spleen (**G**) of tumour-bearing C57BL/6 mice at day 16 post MC38 cell inoculation. ILCs were identified as in **E**. Each dot represents one mouse, closed circle for females. Data show mean ± s.e.m and are pooled from 2 independent experiments with 6 mice/group/experiment. Statistical analyses were performed using unpaired Student's *t* tests and *p* values are indicated.

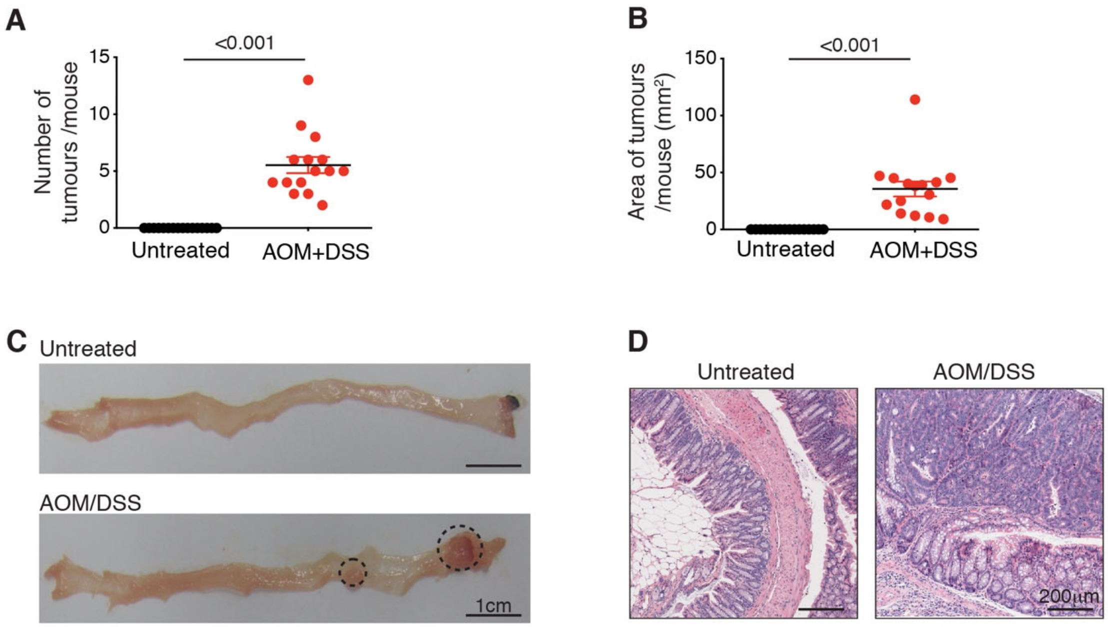

Figure S2. Incidence of CRC tumours in AOM DSS-treated mice. A-**B.** Number (**A**) and area of tumours (mm^2, **B**) per mouse in C57BL/6 mice treated or not with AOM+DSS. Each dot represents one mouse, closed circle for females. Data show the mean ± s.e.m (*n*=2-3 mice/experiment) pooled from four independent experiments. Statistical differences were analysed using the Mann-Whitney nonparametric test. *p* values are indicated. **C**. Representative whole mount images of colons from C57BL/6 mice treated, or untreated, with AOM+DSS. Images are representative of 15-16 mice/genotype analysed in four separate experiments. Dotted lines indicate tumour size and location. Scale bar, 1 cm. **D**. Representative images of colon sections stained with Hematoxylin & Eosin which were collected from naïve and AOM+DSS treated C57BL/6 mice. Images are representative of >10 mice/genotype. Scale bar, 200 μm.

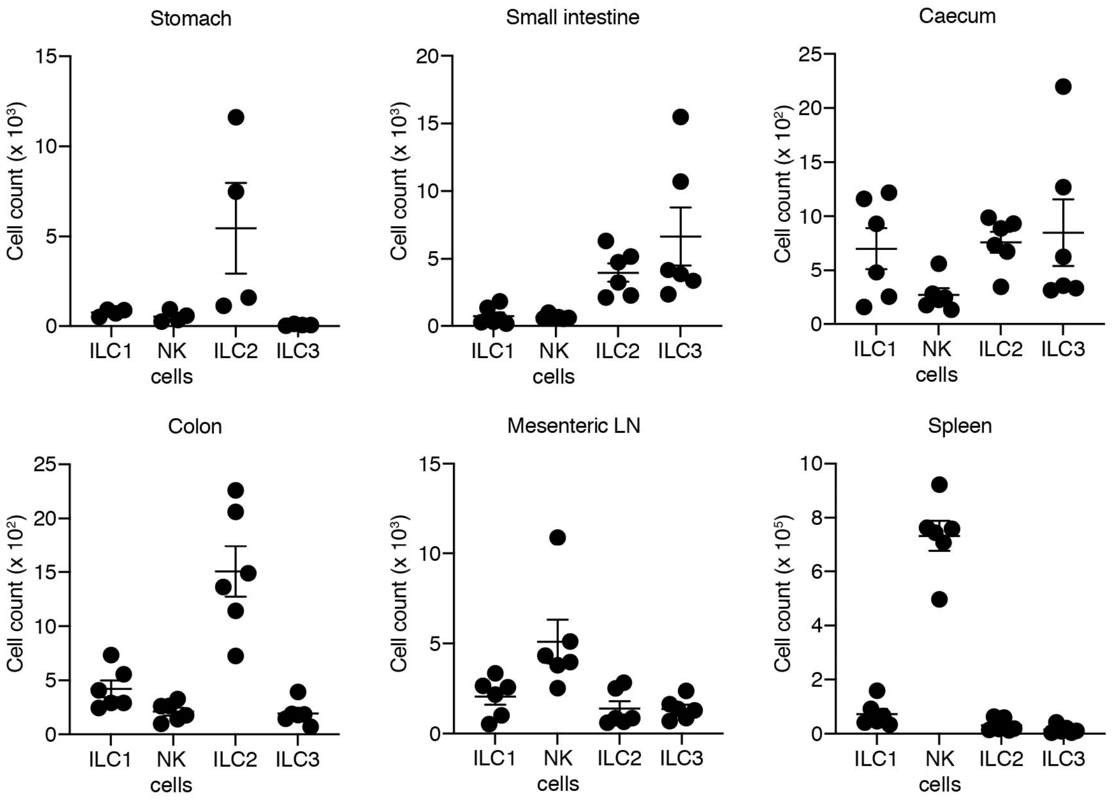

Figure S3. ILC composition within the intestinal tract, mesenteric lymph nodes and spleen of untreated C57BL/6 mice. Enumeration of the ILC subsets in the lamina propria of the stomach, small intestine, caecum and colon as well as in the mesenteric lymph nodes (LN) and spleen of naive C57BL/6 mice. ILC1 were identified as follow: $CD45^+CD3^-$ $NK1.1^+NKp46^+Eomes^-$; NK cells as $NK1.1^+NKp46^+Eomes^+$; ILC2 as $CD127^+GATA3^+$; and ILC3 as $CD127^+ROR\gamma t^+$. Data show the mean $\pm$ s.e.m. Data are pooled from three independent experiments (*n*=2 mice/experiment) except for stomach (one experiment in which each data point represents the average of three stomachs pooled together). Statistical differences are reported in Table S2.

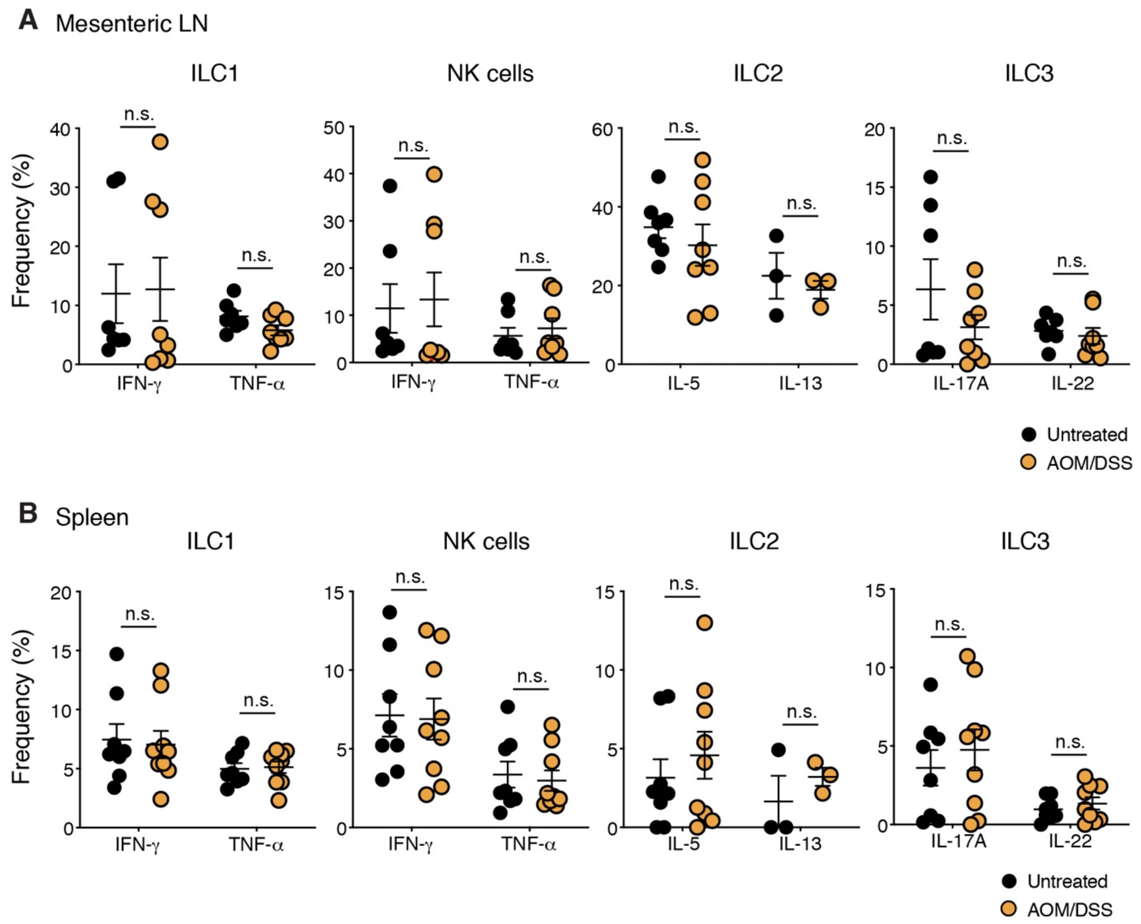

Figure S4. The ILC cytokine production in spleen and mesenteric lymph nodes is not influenced during CAC progression. A-**B**. Intracellular staining flow-cytometric analyses of TNF-α, IFN-γ, IL-5, IL-13, IL-17A and IL-22 cytokine production by ILC1, NK cells, ILC2 and ILC3 isolated from the mesenteric lymph nodes (**A**) and spleens (**B**) after short-term restimulation with PMA, ionomycin in the presence of Golgi Stop and Golgi Plug. Cells were stimulated for 4 hours before staining. Frequency of cytokine-producing ILC isolated from the mesenteric lymph nodes (**A**) and spleen (**B**) of untreated and AOM DSS-treated mice. Each dot represents one mouse. Data show the mean ± s.e.m. IL-13 data is from one experiment, all other data is pooled from four independent experiments (*n*=2-3 mice/genotype/experiment). Statistical differences were analysed using the Mann-Whitney nonparametric test. n.s., not significant.

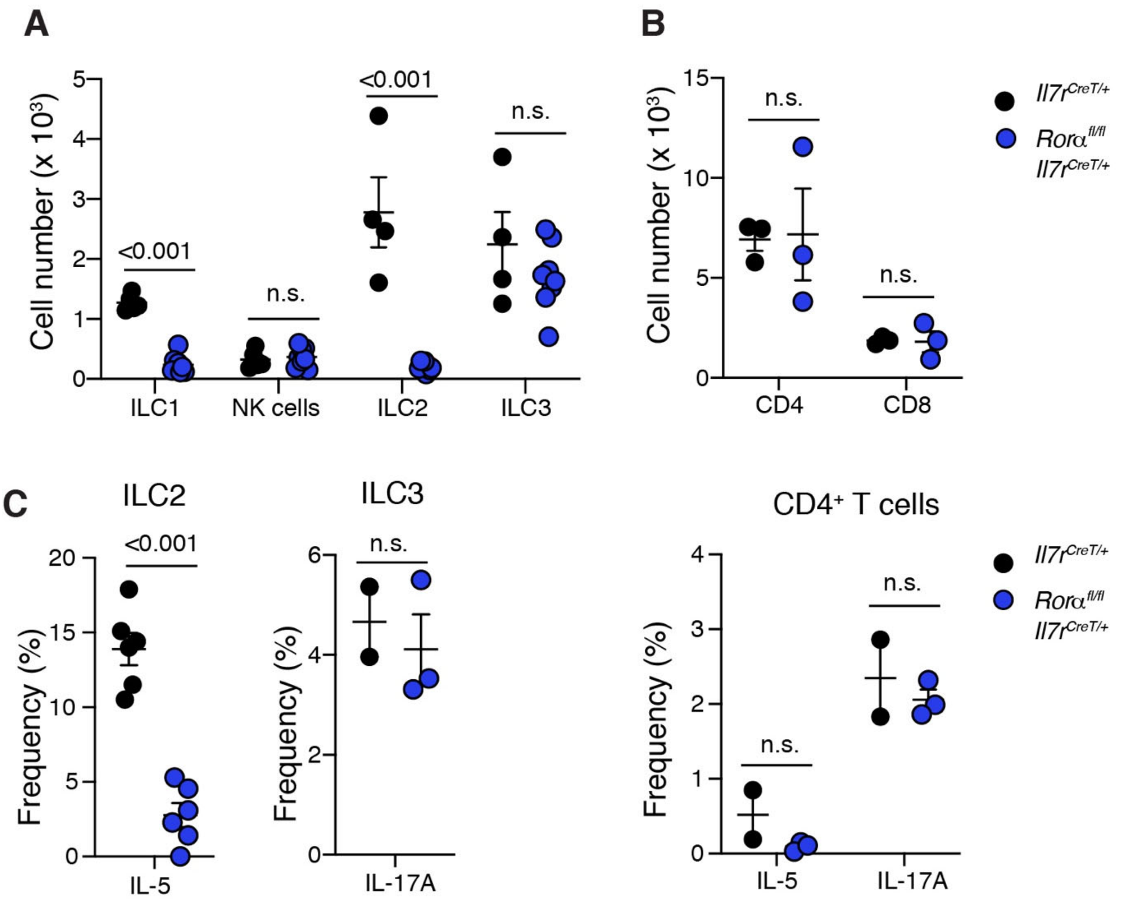

Figure S5. Deletion of RORα results in loss of ILC1 and ILC2 but not T cells or other ILC subsets at steady-state. A-**B**. Enumeration of ILC (**A**) and CD4⁺ and CD8⁺ T cell (**B**) subsets isolated from the colon of naïve 8 weeks old *Il7r*$^{Cre\ T/+}$ and *Rorα*$^{fl/fl}$*Il7r*$^{Cre\ T/+}$ mice. Data show the mean ± s.e.m. ILC2 data is pooled from two independent experiments, ILC3 and T cell data are from one experiment (n=2-4 mice/genotype/experiment). **C**. Frequency of IL-5 and IL-17A cytokine producing ILC2, ILC3 and CD4⁺ T cells. Data show the mean ± s.e.m. ILC2 data is pooled from two independent experiments, ILC3 and CD4⁺ T cell data are from one experiment (n=2-4 mice/genotype/experiment). **A**-**C**. Each dot represents one mouse. Statistical significance was determined using an unpaired Student's t test p values are indicated. n.s., not significant.

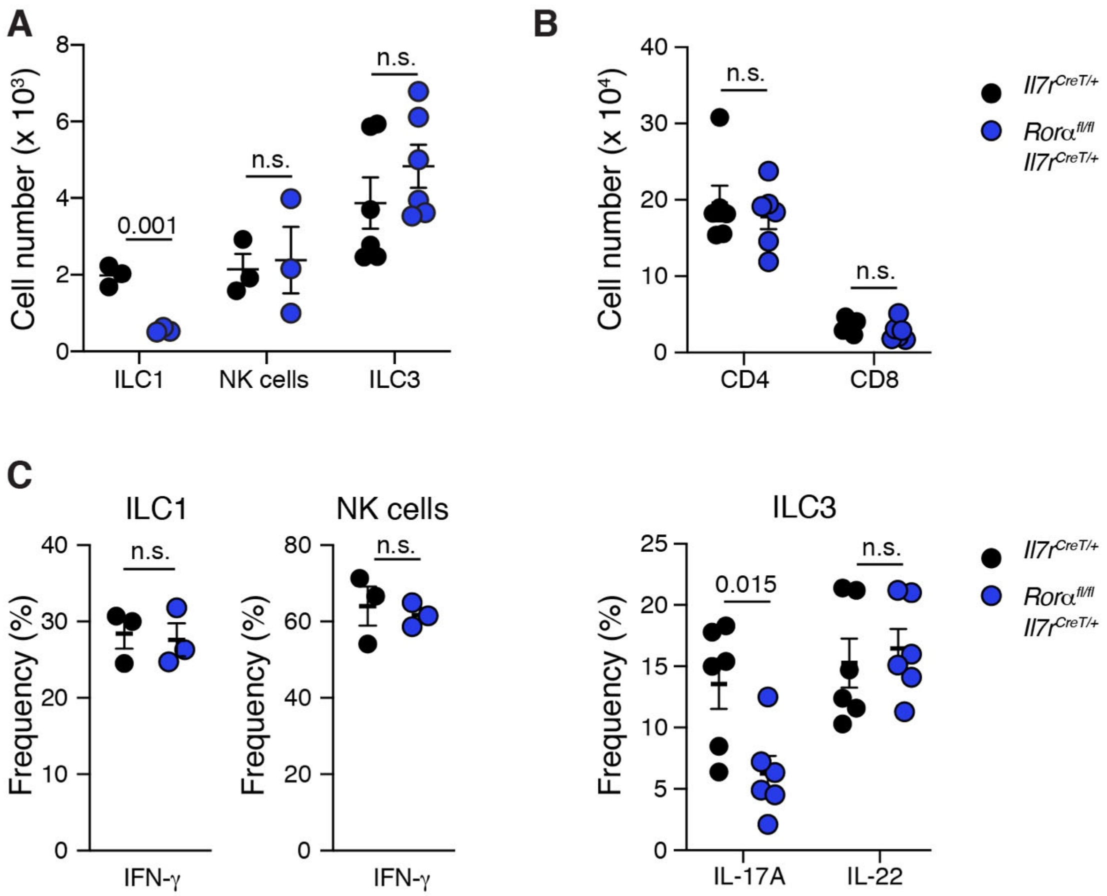

Figure S6. Deletion of RORα results in loss of ILC1 but not cytokine production or other lymphoid subsets during CAC. A. Enumeration of non-ILC2 ILC subsets isolated from the colon of AOM+DSS treated *Il7r*$^{Cre\ T/+}$ and *Rorα*$^{fl/fl}$*Il7r*$^{Cre\ T/+}$ mice. Data show the mean ± s.e.m. ILC3 data is pooled from two independent experiments, ILC1 and NK cell data are from one experiment (*n*=3 mice/genotype/experiment). **B**. Number of colonic CD4^{+} and CD8^{+} T cells of AOM+DSS treated *Il7r*$^{Cre\ T/+}$ and *Rorα*$^{fl/fl}$*Il7r*$^{Cre\ T/+}$ mice. Data show the mean ± s.e.m and are pooled from two independent experiments (*n*=3 mice/genotype/experiment). **C**. Frequency of IFN-γ cytokine producing ILC1 and NK cells and IL-17A/IL22 producing ILC3. Data show the mean ± s.e.m. ILC3 data is pooled from two independent experiments, ILC1 and NK cell data is from one experiment (*n*=2-4 mice/genotype/experiment) **A**-**C**. Each dot represents one mouse. Statistical significance was determined using an unpaired Student's *t* test *p* values are indicated. n.s., not significant.

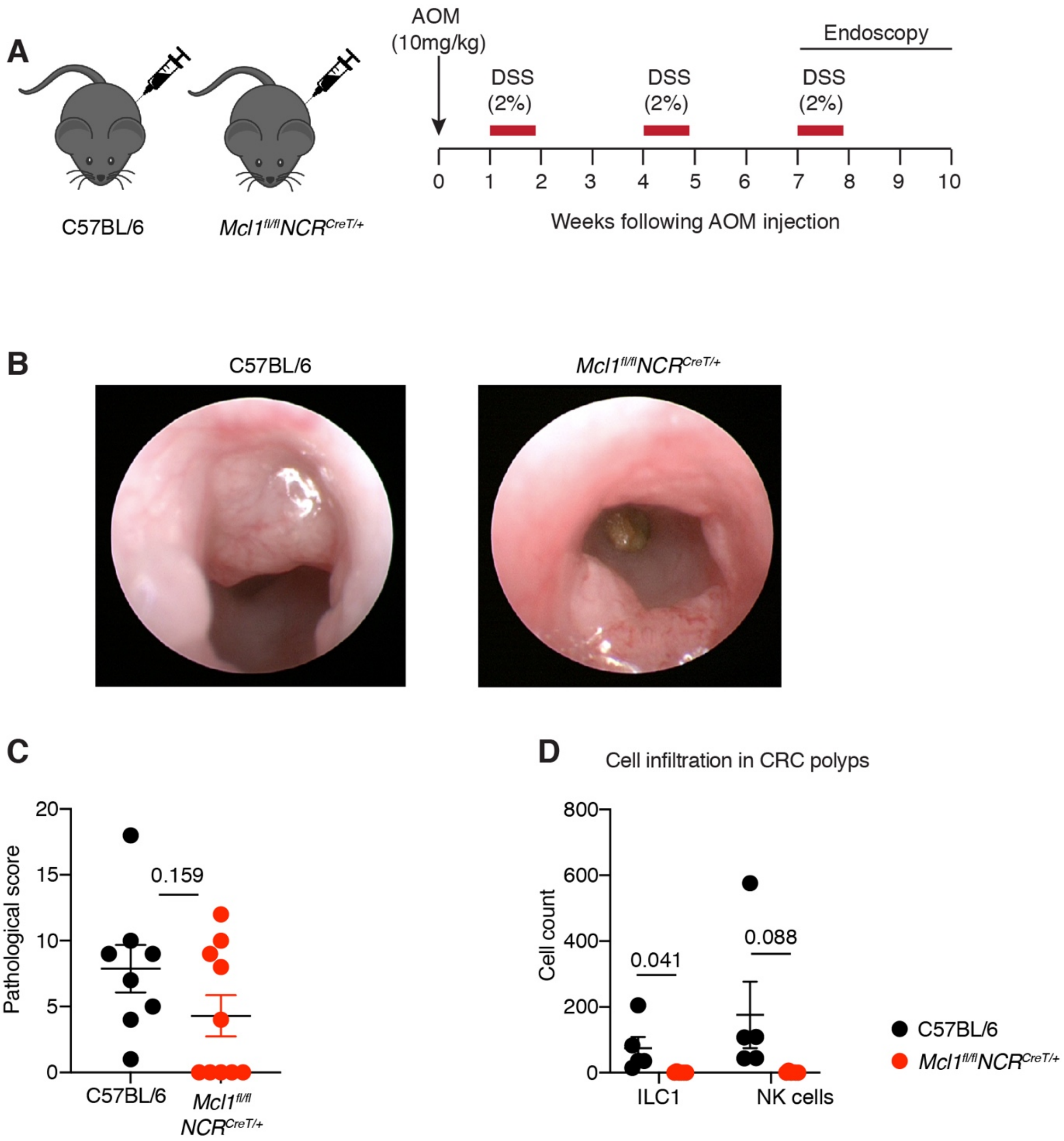

Figure S7. Loss of ILC1 and NK cells does not impact CAC development. A. Schematic illustration of the AOM/DSS treatment protocol. C57BL6 and *Mcl1*$^{fl/fl}$*NCR*$^{CreT/+}$ mice injected with AOM (10mg/kg, i.p) followed by three 5-day cycles of 2% (w/v) DSS *ad libitum* in their drinking water separated by two weeks of normal water between each cycle. Colonic tumours developed between 7-10 weeks after the initial commencement of treatment. **B**. Endoscopic images of AOM/DSS-treated C57BL6 and *Mcl1*$^{fl/fl}$*NCR*$^{CreT/+}$ mice. Images are representative of 8 to 10 mice/genotype. **C**. Pathological score of colons from C57BL6 or *Mcl1*$^{fl/fl}$*NCR*$^{CreT/+}$ mice treated with AOM+DSS. **D**. FACS enumeration of group 1 innate lymphoid cell (ILC1 – Eomes$^-$CD49a$^+$; NK cells – Eomes$^+$CD49a$^-$) infiltration in colons of C57BL/6 and *Mcl1*$^{fl/fl}$*NCR*$^{CreT/+}$ mice treated with AOM+DSS. Each dot represents one mouse. Data show the mean $\pm$ s.e.m (n=5-10 mice/genotype). Statistical differences were analysed using unpaired Student's t test. p-values are indicated.

Table S1. Longitudinal, cross-sectional and survival analyses from MC38 tumour-bearing C67BL/6, *Rag1*$^{-/-}$ and *Rag2*$^{-/-}$*γc*$^{-/-}$ for TumGrowth analysis.

Longitudinal analysis

Type II ANOVA

	**F test (KR)**	**F test (S)**	**LR test**	**Wald test**
Time x Treat	88.64 (d.f.=2/63.7), *p*<<0.0001	88.76 (d.f.=2/74.6), *p*<<0.0001	75.49 (d.f.=2), *p*<<0.0001	177.52 (d.f.=2), *p*<<0.0001
Time	527.42 (d.f.=1/39.7), *p*<<0.0001	527.64 (d.f.=1/36.7), *p*<<0.0001	101.58 (d.f.=1), *p*<<0.0001	527.64 (d.f.=1), *p*<<0.0001
Treat	9.17 (d.f.=2/34.1), *p*<0.0007	9.24 (d.f.=2/401.0), *p*<0.0001	15.82 (d.f.=2), *p*<0.0004	18.48 (d.f.=2), *p*<<0.0001

Selected pairwise comparisons

p-value adjustment: Holm

**Largest**	**Smallest**	**Contrast**	**Df**	***p*-value**	***p*-value adjusted**
***Rag1*$^{-/-}$**	**C57BL/6**	0.089 [0.072;0.106]	55.18	<0.0001	<0.0001
***Rag2*$^{-/-}$*γc*$^{-/-}$**	**C57BL/6**	0.099 [0.082;0.117]	63.02	<0.0001	<0.0001
***Rag2*$^{-/-}$*γc*$^{-/-}$**	**Rag1$^{-/-}$**	0.010 [-0.008;0.029]	81.48	0.2661	0.2661

Cross-sectional analysis

ANOVA

	**Likelihood ratio test**	**F test**	**Kruskall-Wallis**
Treat	48.12 (d.f.=2), *p*<<0.0001	61.16 (d.f.=2), *p*<<0.0001	25.40 (d.f.=2), *p*<<0.0001

Selected pairwise comparisons

p-value adjustment: Holm

**Group 1**	**Group 2**	**Difference**	***p*-value**	***p*-value adjusted**	**Wilcox *p*-value**	**Wilcox *p*-value adjusted**
***Rag1*$^{-/-}$**	**C57BL/6**	41.917 [28.333;55.500]	<0.0001	<0.0001	<0.0001	0.0001
***Rag2*$^{-/-}$*γc*$^{-/-}$**	**C57BL/6**	62.958 [47.417;78.500]	<0.0001	<0.0001	<0.0001	0.0001
***Rag2*$^{-/-}$*γc*$^{-/-}$**	**Rag1$^{-/-}$**	21.042 [2.089;39.995]	0.0097	0.0097	0.0260	0.0260

Survival analysis (*Rag1*$^{-/-}$ as a reference)

ANOVA

	**Likelihood ratio test**	**Wald test**	**Log-Rank test**
Treat	46.29 (d.f.=2), *p*<<0.0001	4.38 (d.f.=2), *p*<0.1120	36.97 (d.f.=2), *p*<<0.0001

Hazard ratios

p-value adjustment: Holm

**Covariate**	**Hazard ratio**	***p*-value**	***p*-value adjusted**	**Log-Rank *p*-value**	**Log-Rank *p*-value adjusted**
**C57BL/6**	-Inf [-Inf;Inf]	0.8440	0.8440	<0.0001	<0.0001
***Rag2*$^{-/-}$*γc*$^{-/-}$**	2.556 [1.057;6.183]	0.0373	0.0745	0.0313	0.0313

Table S2. ANOVA analysis of ILC subsets in gastrointestinal organs at steady-state.

Comparison	Stomach	Small intestine	Caecum	Colon	Mesenteric LN	Spleen
ILC1 *vs.* NK	>0.9999	0.0173	0.3003	0.5646	0.2650	0.4347
ILC1 *vs.* ILC2	0.7133	0.0042	>0.9999	0.5181	>0.9999	>0.9999
ILC1 *vs.* ILC3	0.2697	0.0766	>0.9999	0.3968	>0.9999	0.1649
NK *vs.* ILC2	0.2697	>0.9999	0.0539	0.0042	0.0105	0.0151
NK *vs.* ILC3	0.7133	0.0225	0.1335	>0.9999	0.0219	0.0004
ILC2 *vs.* ILC3	0.0022	>0.9999	>0.9999	0.0023	>0.9999	>0.9999

Underlined numbers represent *p*-values of comparisons with statistically significant difference.

Table S3. ILC2 gene signature.

Feature	Weight	Feature	Weight	Feature	Weight
IL2	0.206869	*FLT3LG*	0.00111423	*IFI16*	0
CCR4	0.0863433	*RUNX3*	0.00089958	*EWSR1*	0
ECSIT	0.0735243	*SMAD3*	0.00074262	*ITK*	0
IL10RA	0.055715	*CD53*	0.00071255	*TNFSF10*	0
LRRN3	0.0531323	*CD44*	0.00071142	*CYFIP2*	0
IL17RB	0.0462906	*JAK2*	0.00065083	*ILF3*	0
ITGAE	0.0461633	*CCR1*	0.00061332	*CYLD*	0
IL1RL1	0.0404291	*STAT1*	0.00060124	*MAP3K7*	0
KLRG1	0.0362019	*CD81*	0.00059675	*IFITM2*	0
CSF2	0.0236164	*IRF4*	0.00056124	*PIK3CD*	0
CD74	0.0232307	*TNFSF14*	0.00055953	*NUP107*	0
GATA3	0.0224158	*IL7R*	0.0005343	*ITGAL*	0
TNFRSF4	0.0218217	*HAVCR2*	0.00052931	*TFRC*	0
CD27	0.0206863	*EGR1*	0.00050366	*TRAF6*	0
PRKCE	0.0206544	*LYN*	0.00046362	*IRF7*	0
OSM	0.0199132	*IRAK1*	0.00046162	*CD63*	0
PDCD1	0.0154011	*MAPK1*	0.00034388	*IFI35*	0
FOS	0.00974204	*LY9*	0.00033311	*CLU*	0
POU2F2	0.00847504	*BCL2L1*	0.00029227	*CD28*	0
CASP1	0.00750729	*RELA*	0.00026883	*FAS*	0
IL15RA	0.00635427	*CDKN1A*	0.00022799	*ATG5*	0
JAK3	0.00620136	*ABCB1*	0.00019413	*CD37*	0
CD3EAP	0.00594586	*ATF2*	0.00017671	*TYK2*	0
NFKBIA	0.00586913	*REPS1*	0.00016272	*RIPK2*	0
ISG15	0.00576078	*IL2RA*	0.000162	*LAMP2*	0
TRAF3	0.00471944	*CD96*	0.00012582	*ST6GAL1*	0
ADA	0.00421412	*YTHDF2*	0.00012306	*IRF1*	0
NCF4	0.0038821	*ATG16L1*	0.00012085	*DDX58*	0
IL2RG	0.00353999	*ANP32B*	0.00010744	*ENTPD1*	0
LIF	0.00337071	*TBK1*	0.00010289	*STAT4*	0
CASP8	0.00319852	*STAT3*	8.36E-05	*GTF3C1*	0
IFNAR1	0.0030248	*ENG*	7.33E-05	*SMAD2*	0
GPI	0.00229326	*PSEN2*	3.87E-05	*MAF*	0
C1QBP	0.00215396	*ANXA1*	2.94E-05	*CASP3*	0
CHUK	0.00170722	*PTPRC*	6.07E-06	*INPP5D*	0
CXCR6	0.00146083	*TXNIP*	0	*MAPK3*	0
IL2RB	0.00144123	*CD2*	0	*PTGS2*	0

TNFRSF18	0.00133321	*KIT*	0	*IL21R*	0
PLAUR	0.00113747	*CD84*	0	*NT5E*	0
**Feature**	**Weight**	**Feature**	**Weight**	**Feature**	**Weight**
NFATC1	0	*TOLLIP*	0	*IL18RAP*	-0.00238245
IRF2	0	*BCL10*	0	*CD164*	-0.00244962
NOTCH1	0	*IL17RA*	0	*BST2*	-0.0029268
MAP4K2	0	*IFNAR2*	0	*STAT6*	-0.00312513
MAP2K4	0	*IL12RB1*	-3.20E-06	*MAPK14*	-0.00335746
BATF	0	*ZAP70*	-3.02E-05	*TNFSF4*	-0.00347132
NFATC3	0	*IFNGR1*	-4.11E-05	*TCF7*	-0.00397718
ATG10	0	*BAX*	-4.31E-05	*CD3G*	-0.0062911
MAP3K5	0	*HLA-DQA1*	-4.75E-05	*NRP1*	-0.00657612
PSEN1	0	*TNFRSF9*	-4.95E-05	*IL18R1*	-0.00660455
ICAM1	0	*TRAF2*	-6.00E-05	*ITGA5*	-0.0070242
STAT5B	0	*ATG7*	-7.26E-05	*TXK*	-0.00755759
STAT2	0	*ICOS*	-8.01E-05	*IRAK2*	-0.00774614
MAPK8	0	*CSF1*	-8.99E-05	*NFATC2*	-0.00803036
TNFRSF11A	0	*CCR2*	-0.00010368	*TNFAIP3*	-0.00854714
MAVS	0	*TFE3*	-0.00011479	*TIGIT*	-0.0087403
CREB1	0	*IL16*	-0.00013147	*TICAM1*	-0.00895162
IRF3	0	*PECAM1*	-0.00013353	*IRF8*	-0.0100038
IKBKE	0	*BCL2*	-0.00013441	*ISG20*	-0.010305
PRKCD	0	*CD48*	-0.00017676	*TNFRSF1A*	-0.0115377
THBS1	0	*TANK*	-0.00020269	*MEF2C*	-0.0119517
TNFRSF1B	0	*LRP1*	-0.00021933	*IRF5*	-0.0119688
EP300	0	*APP*	-0.00024874	*SLAMF1*	-0.0121617
SIGIRR	0	*CCL5*	-0.00027321	*RORC*	-0.0132505
IL11RA	0	*CCND3*	-0.00029067	*LGALS3*	-0.0139224
FADD	0	*ITGB2*	-0.00033562	*MYD88*	-0.0146959
IGF2R	0	*SELPLG*	-0.00044161	*CTSH*	-0.0150875
PSMD7	0	*CTSS*	-0.0004603	*SPN*	-0.0154769
IKBKB	0	*ATF1*	-0.00049572	*LCK*	-0.0157092
CEBPB	0	*MAP3K1*	-0.00050973	*JAK1*	-0.0157398
CD47	0	*UBC*	-0.00084905	*CXCR4*	-0.0213308
ABL1	0	*DUSP4*	-0.00110906	*IGF1R*	-0.0239232
REL	0	*CD247*	-0.00113715	*DUSP6*	-0.0245169
NLRC5	0	*NLRP3*	-0.00119608	*DOCK9*	-0.0253211
MAP2K1	0	*LCP1*	-0.00134211	*IL1R1*	-0.027627
PIK3CG	0	*ITGA6*	-0.00148169	*CTSW*	-0.0294796
HLA-DMA	0	*CD3D*	-0.00154358	*CCR7*	-0.0352911
MAP2K2	0	*TNFSF11*	-0.00176661	*FCER1G*	-0.0356495
TP53	0	*CD83*	-0.00186132	*TNFSF8*	-0.107785
ATM	0	*IL6R*	-0.00216832	*CCR6*	-0.156758
CARD11	0	*IRAK4*	-0.00236336	*KLRB1*	-0.17907
				KLRC1	-0.208495
